# Co-Morbidities as Predictors of Airflow Limitation among Smokers in England

**DOI:** 10.3390/pharmacy6020045

**Published:** 2018-05-22

**Authors:** Reem Kayyali, Yusur Hassan, Iman Hesso, Roshan Siva

**Affiliations:** 1 School of Life Sciences, Pharmacy and Chemistry, Kingston University London, Penrhyn Road, Kingston Upon Thames KT1 2EE, UK; k1039336@kingston.ac.uk (Y.H.); hesso.iman@gmail.com (I.H.); 2 Croydon Health Services NHS Trust, Lennard Road, Croydon CR7 7YE, UK; roshan.siva@nhs.net

**Keywords:** Chronic Obstructive Pulmonary Disease (COPD), airflow limitation, co-morbidities, smoking, primary care

## Abstract

The prevalence of co-morbidities among patients with Chronic Obstructive Pulmonary Disease (COPD) is well documented in the literature. Therefore, this pilot study aimed to identify whether co-morbidities screening would enhance COPD case-finding. Smoking patients were approached at Croydon University Hospital and two local community pharmacies (CPs). Their co-morbidities, respiratory symptoms, smoking pack-years and exercise capacity were collected. Airflow limitation was determined using handheld spirometry (COPD-6) device. The prevalence of airflow limitation was 42% (*n* = 21/50). The main identified predictors of airflow limitation were: co-morbidities (OR = 9, CI: 1.04–77.81, *p* = 0.025), respiratory symptoms (OR = 33.54, CI: 1.06–11.77, *p* = 0.039) and smoking history of ≥20 pack-years (OR = 3.94, CI: 1.13–13.64, *p* = 0.029). CPs were the main location for case-finding. This study demonstrated the need to screen for co-morbidities for COPD case-finding within CPs.

## 1. Introduction

Chronic Obstructive Pulmonary Disease (COPD) is underdiagnosed in many countries [1]. In the UK, 3 million people are estimated to have COPD with only 900,000 diagnosed [2]. Smoking is the main risk factor for COPD development [1,3,4]. Unfortunately, patients are usually in a more advanced stage when the disease is brought to clinical attention [3]. The prevalence of co-morbidities such as diabetes, cardiovascular diseases, anxiety and depression among COPD patients is well documented in the literature [5]. These co-morbidities markedly affect health outcomes in COPD [5] including mortality and morbidity [6]. Increasing evidence in the literature suggests a link between COPD and these co-morbidities which are considered to be a part of the commonly prevalent non-pulmonary sequelae of the condition due to the presence of chronic systemic inflammation [7,8].

In Croydon, the proposed estimated prevalence of COPD is 4.6%. However, general practice records indicate a prevalence of 0.7%, demonstrating under-diagnosis [9]. The cost of COPD hospital admission is estimated to be £3695 [9] compared to the national average cost in England of £1960 [10]. Hence, a COPD strategy was launched in Croydon to enhance diagnosis, support to patients and reduce hospital admissions [9].

This pilot study aimed to identify whether co-morbidities detection could enhance COPD diagnosis using handheld spirometer (COPD-6 device) in chronic smokers in Croydon.

## 2. Materials and Methods

### 2.1. Participants and Recruitment

Patients were approached at Croydon University Hospital (CUH) and two community pharmacies (CPs) in Croydon, from January to April 2016. Patients aged between 35 and 70, who had smoking history of more than 10 pack-years and were medically fit to perform the COPD-6 test were included. Patients with any of the following (contraindications for COPD-6 test) were excluded: uncontrolled blood pressure, aneurysm (not treated), ear infection or perforation of the ear drum, eye problems (glaucoma), dementia, pregnancy, current or recent respiratory infection and/or taking antibiotics or prednisolone within the last 6 weeks, unwell (nausea, vomiting or pain); or if they suffered from any of the following within the last 3 months: collapsed lung (pneumothorax), haemoptysis of unknown cause, myocardial infarction or unstable cardiac condition, abdominal, chest, ear or eye surgery, blood clot on the lung (pulmonary embolus), stroke. Patients were also excluded if they were already diagnosed with asthma or COPD.

### 2.2. Questionnaire

Data collection was conducted using a short paper-based questionnaire. The questionnaire was designed to elicit information about: patient’s details, respiratory symptoms, pack-years smoking history, medical history, COPD-6 test and history of co-morbidities (Appendix A). In addition, patients’ exercise capacity was assessed using the Medical Research Council (MRC) breathlessness scale [11].

Following the completion of the questionnaire, patients were asked to perform the forced expiratory manoeuvre for at least six seconds using the handheld spirometer COPD-6 device. Patients were asked to do at least two acceptable measurements and those with ratio of FEV1/FEV6 < 0.75 were considered to have possible airflow obstruction/limitation [12].

### 2.3. Data Analysis

The data was entered into Microsoft Excel. Odds ratios were calculated to measure the association of a variable with airflow limitation. Barnard’s Exact Test was used to test the statistical significance difference of variables and of the odds ratio not equalling 1. The level of significance was set at *P* < 0.05.

### 2.4. Ethical Consideration

Ethical approval was granted by the Kingston University Delegated Research Ethics Committee (Reference No. 1213/045).

## 3. Results

### 3.1. Participants’ Characteristics

**Figure 1:**
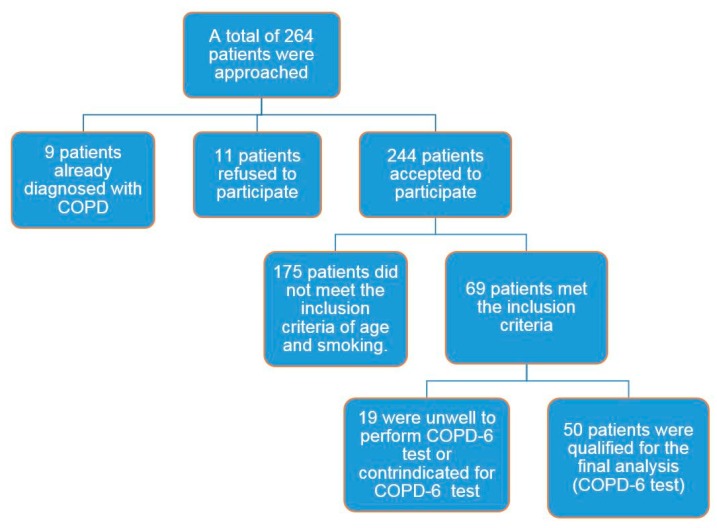
Participants recruitment: total number of patients approached during the study (*n* = 264).

Total of 264 patients were approached; 214 patients were excluded and 50 patients were included and performed the COPD-6 test (Figure 1). Participants’ characteristics are summarised in Table 1.

### 3.2. COPD-6 Screening

42% of patients (*n* = 21/50) had a ratio of FEV1/FEV6 < 0.75 and considered to have possible airflow limitation (Table 1). The majority of those with airflow limitation had an MRC breathlessness score of grade 3 or above (71%, *n* = 15/21) (*p* = 0.039). Besides smoking, the only demographic factor that was significantly different between the two groups was the presence of co-morbidities (*p* = 0.025). The co-morbidities present in each group are summarised in Table 2.

Patients were approached at the main outpatient department and the smoking cessation clinic at CUH, in addition to CPs. Considering the time spent in each location, 1 patient per day was screened positive in CPs compared to 1 patient per 3 days and 4 days in the main outpatient department and smoking cessation clinic respectively.

### 3.3. Predictive Factors of Airflow Limitation

The median smoking history was 25 pack-years among patients having airflow limitation, with 67% (*n* = 14/21) still actively smoking. Smoking history of ≥20 pack-years had an odds ratio of 3.94 (CI: 1.13–13.64) in predicting airflow limitation.

Co-morbidities were prevalent in most patients (95%, *n* = 20/21) with airflow limitation. Nearly two-third of the patients (62%, *n* = 13/21) had two or more co-morbidities. The odds to have airflow limitation in patients with co-morbidities was 9 (CI: 1.04–77.81).

The majority of patients with airflow limitation had early respiratory symptoms. The presence of such symptoms had an odds ratio of 3.54 (CI: 1.06–11.77) in predicting airflow limitation (Table 3). Only 26.6% (*n* = 4/15) have reported the symptoms to their general practitioner (GP); two out of the four who reported their symptoms have performed spirometry.

## 4. Discussion

In this study, the prevalence of airflow limitation was 42%. However, all patients that were identified to have airflow limitation will need to perform standard spirometry test to confirm diagnosis. In Ching et al. [13] study, the prevalence of airflow limitation using the handheld spirometry was 10.6%, but when airflow limitation was confirmed with spirometry, the prevalence was 6%. Following the analogy of Ching et al. [13] study, the corrected prevalence of airflow limitation would be reduced from 42% to 23.8%. Consequently, 12 of the 21 patients who screened positive with the handheld spirometry will be confirmed to have COPD.

CPs were found to be suitable premises for COPD screening in the current study. The positive role of community pharmacists in early detection/screening of COPD has been already emphasized in the literature [14,15]. A pilot study in the UK showed that community pharmacy COPD-screening could save the NHS £264 million [15].

Parameters such as age, smoking history and early respiratory symptoms, have been already identified and used to aid in COPD screening [16]. However, the current study identified co-morbidities as an additional significant predictor (*p* = 0.025) to COPD detection/screening. Despite that the majority of patients with airflow limitation had early respiratory symptoms. Yet only four patients reported this to their GP, and only two performed spirometry. This highlights the need to enhance public awareness about these symptoms, as a comprehensive approach to their management. It also highlights the need to raise awareness among healthcare professionals about the importance of investigating these reported symptoms and performing spirometry testing as appropriate.

The current study is a pilot study with small sample size which limits the generalisability of the results. In addition, the study employed a self-administered questionnaire thus the data collected was self-reported which is another limitation.

## 5. Conclusions

The current study, although limited by a small sample size, demonstrated the need to consider co-morbidities as a primary screening parameter for COPD in the efforts of Croydon to optimise early diagnosis. The study also highlighted that CPs can provide a suitable venue for early screening of COPD.

## Figures and Tables

**Table 1:** Participants demographics, clinical characteristics and airflow limitation (*n* = 50).

Variables	No Airflow Limitation (*n* = 29)	Have Airflow Limitation (*n* = 21)	*P*-Values
Age, years (median)	53	56	
Age ≥ 60, *n* (%)	8 (28%)	9 (43%)	0.336
Age < 60, *n* (%)	21 (72%)	12 (57%)	
Female gender, *n* (%)	19 (66%)	11 (52%)	0.371
Male gender, *n* (%)	10 (34%)	10 (48%)	
Smoking history, pack-years	-	-	
<20, *n* (%)	16 (55%)	5 (24%)	0.029
≥20, *n* (%)	13 (45%)	16 (76%)	
Respiratory symptoms, *n* (%) MRC breathlessness grade ≥3	12 (41%)	15 (71%)	0.039
Presence of co-morbidities, *n* (%)	20 (69%)	20 (95%)	0.025

**Table 2:** Breakdown of co-morbidities within the recruited sample.

Co-Morbidity	No Airflow Limitation (*n* = 29)	Have Airflow Limitation (*n* = 21)
Hypertension (HTN), *n* (%)	4 (14%)	9 (43%)
Cardiovascular diseases, *n* (%)	3 (10%)	5 (24%)
Hyperlipidaemia, *n* (%)	7 (24%)	4 (19%)
Depression, *n* (%)	5 (17%)	10 (48%)
Diabetes, *n* (%)	4 (14%)	4 (19%)
Osteoporosis, *n* (%)	2 (7%)	3 (14%)
Peripheral Vascular Disease (PVD), *n* (%)	6 (21%)	3 (14%)

**Table 3:** Predictors of airflow limitations in smokers.

Predictors of Airflow Limitation in Smokers (*n* = 50)	
Independent variables	Odds ratio (95% CI)
Age ≥ 60	1.97 (0.600–6.45)
Gender (male)	1.72 (0.55–5.45)
Smoking history ≥ 20 pack-years	3.94 (1.13–13.64)
Presence of respiratory symptoms	3.54 (1.06–11.77)
Presence of co-morbidities	9 (1.04–77.81)

## Appendix A. Study Questionnaire

**Co-morbidity screening sheet**

Name:	
Date Of Birth:	
Age:	
Phone number:	
Ward:	

**Background:**

Have you noticed increasing breathlessness, chronic cough, sputum production, or wheeze over the last few years? Y/N

Having noticed these symptoms. Did you seek medical advice? Y/N

When medical advice sought, were you asked to perform spirometry? Y/N

Any comments?

Height:	
MRC:	1/2/3/4/5
Smoking status:	Y/N
Pack years:	

**Comorbidity:**

Ischaemic Heart Disease (IHD)/Angina: Y/N

Pulmonary Hypertension: Y/N

Peripheral Vascular Disease (PVD): Y/N

Diabetes: Y/N

Cardiovascular Disease (CVD): Y/N

Osteoporosis: Y/N

Hyperlipidaemia: Y/N

Depression: Y/N

Others:

**Medical history:**

Current or suspected respiratory infection	Y/N
Any antibiotics/prednisolone within the last 6 weeks	Y/N
Aneurysm (not treated)	Y/N
Unwell today, i.e., nausea, vomiting or pain	Y/N
Uncontrolled blood pressure	Y/N
Communication problems, i.e., dementia	Y/N
Ear problems, i.e., ear infection	Y/N
Eye problems, i.e., glaucoma	Y/N
Pregnancy	Y/N

**Any of the following within the last 3 months:**

Collapsed lung	Y/N
Haemoptysis of unknown cause	Y/N
Myocardial infarction or unstable heart condition	Y/N
Abdominal, chest, ear or eye surgery	Y/N
PE or Blood clot on the lung	Y/N
Stroke	Y/N

Fit for spirometry? Y/N.

**COPD6 Test:**

	FEV1	FEV1% Pred	FEV6	FEV6% Pred	Ratio
Effort 1					
Effort 2					
Effort 3					

Refer to spirometry team for follow up? Y/N

Any comments:

PATIENT NAME:     SIGNATURE:     DATE:

CLINICIAN NAME:    SIGNATURE    DATE:
